# CTSB is a negative prognostic biomarker and therapeutic target associated with immune cells infiltration and immunosuppression in gliomas

**DOI:** 10.1038/s41598-022-08346-2

**Published:** 2022-03-11

**Authors:** Kaiming Ma, Xin Chen, Weihai Liu, Suhua Chen, Chenlong Yang, Jun Yang

**Affiliations:** 1 Department of Neurosurgery, Peking University Third Hospital, 49 North Garden Rd, Haidian District, Beijing, 100191 China; 2 Center for Precision Neurosurgery and Oncology of Peking University Health Science Center, Beijing, China

**Keywords:** Diagnostic markers, Prognostic markers, Cancer, Diagnosis, Prognosis, Therapeutics, Cancer, Surgical oncology, Cancer therapy, CNS cancer, Tumour biomarkers, Transcription

## Abstract

Previous researches have demonstrated the meaning of CTSB for the progress of several tumors, whereas few clues about its immunological characteristic in gliomas. Here we systematically explored its biologic features and clinical significance for gliomas. 699 glioma cases of TCGA and 325 glioma cases of CGGA were respectively included as training and validating cohorts. R software was used for data analysis and mapping. We found that CTSB was remarkably highly-expressed for HGG, IDH wild type, 1p19q non-codeletion type, MGMT promoter unmethylation type and mesenchymal gliomas. CTSB could specifically and sensitively indicate mesenchymal glioma. Upregulated CTSB was an independent hazard correlated with poor survival. CTSB-related biological processes in gliomas chiefly concentrated on immunoreaction and inflammation response. Then we proved that CTSB positively related to most inflammatory metagenes except IgG, including HCK, LCK, MHC II, STAT1 and IFN. More importantly, the levels of glioma-infiltrating immune cells were positively associated with the expression of CTSB, especially for TAMs, MDSCs and Tregs. In conclusion, CTSB is closely related to the malignant pathological subtypes, worse prognosis, immune cells infiltration and immunosuppression of gliomas, which make it a promising biomarker and potential target in the diagnosis, treatment and prognostic assessment of gliomas.

## Introduction

Now gliomas have become the most common type of primary tumors in central nervous system (CNS), comprising 80.8% of the primary malignant tumors and resulting in 88.10% of the deaths in CNS tumors^1^. The current therapies for gliomas include surgical resection, chemotherapy, radiation therapy and some other emerging treatments such as molecular targeted therapy, immunotherapy and tumor treating fields (TTF)^2,3^. Yet the holistic treatment status of gliomas has achieved limited improvements with these therapies, and the therapeutic response of high-grade gliomas (HGG) is still poor^3–5^. More recently, WHO CNS5 has highlight the significance of molecular biomarkers in providing meaningful diagnostic and therapeutic information for gliomas^6^. Enrichment strategies using precise biomarkers will help to improve the current dilemma of glioma treatment^4^. Thus, it is urgent to find more specific molecular biomarkers with clinicopathologic utility closely related to the malignant behavior and immune microenvironment of gliomas.

Cathepsin B (CTSB) is an exclusive multifunctional protein of cysteine protease family in that it has an additional pH sensitive occluding loop which allows it to act as an endopeptidase/exopeptidase depending upon pH^7^. There are three major subtypes of CTSB^8^: main transcript, main transcript lacking exon 2 or main transcript lacking exon 2 and 3. The latter two isoforms were proved as a result of alternative splicing^9,10^, which directly influences the translation rate, sorting and catalytical activity of CTSB^11–14^. CTSB is tightly regulated transcriptionally, post-transcriptionally and post-translationally from biosynthesis to lysosomes^15^, such as transcription level increased, transcription start sites alterations, splice variants adjustments and post-transcriptional modifications via proteolytic processing, glycosylation, inhibition and trafficking^16^. Different subtypes of CTSB have different subcellular localizations, which determine its multiple biological functions^8^. CTSB locates mainly in secretory vesicles, cytoplasm, nucleus, and also binds to plasma membrane or is secreted outside into the extracellular space^15,17^. It can also be secreted without entering the endosomal-lysosomal system^8^. Translocation of CTSB may occur under pathological conditions, for example, it can locate on the cell membrane of cancer cells via binding to annexin A2(ANXA2) tetramers or the caveolae site^18^. The physiological function of CTSB mainly associates with its carboxypeptidase activity, and the endopeptidase activity of CTSB is related to its pathological role in the degradation of extracellular matrix (ECM)^8^. The principal physiological function of CTSB is the lysosomal degradation of different proteins to maintain the stable state of intracellular proteome^15^. CTSB has also been proved to participate in ECM remodeling by degrading the structural components, such as collagen and elastin^17^. It also can affect various intracellular signaling pathways with the processing of several cytokines and chemokines^17^. CTSB plays a key role in many physiological and pathological processes such as cell proliferation, migration, autophagy, antigen presentation, apoptosis^19^, hippocampal dependent memory function^20^, cell differentiation and tumorigenesis^17^.

In terms of pathological processes, CTSB related to arthritis and atherosclerotic vascular diseases^17^, and has also been confirmed to be involved in inflammatory brain diseases and brain aging^21^, memory and psychiatric disorders and neuronal cascade death response after cerebral infarction^22^. CTSB has been regarded as an important biomarker and potential therapeutic target for many cancers^15^, such as pancreatic cancer^23^, gastric cancer^24^, colon cancer^25^, breast cancer^8^, acute myelogenous leukemia^26^, oral squamous cell carcinoma and thyroid cancer^27^. Recent studies have confirmed that CTSB is rarely expressed in normal brain tissues but overexpressed in glioblastoma (GBM) and glioblastoma stem cells (GSCs)^28,29^. The expression level of CTSB in GBM was 6 times higher than that in normal brain tissues^30^. For GBM, CTSB mainly locates in the invasive margins of tumor infiltration and neovascularization^31^. Additionally, it was shown that highly-expressed CTSB can promote the proliferation^32^, migration^33^, adhesion^15^, invasion^18^, anti-apoptosis^34^, tumor angiogenesis^8^ and drug resistance of GBM tumor cells^35^. These make CTSB a possible therapeutic target for gliomas. Yet previous researches about CTSB in gliomas mainly focused on GBM, mostly in vitro, and lacked of comprehensive large-sample clinical analysis, which are inadequate in reflecting the actual role of CTSB regarding the immune microenvironment and tumor heterogeneity of gliomas. More importantly, there were few clues about the role of CTSB in glioma-related immune activities, which greatly limits the understanding of the function and mechanism of CTSB in gliomas and restricts the clinical transformation of CTSB-targeted therapy for gliomas.

In this study, we aimed to comprehensively explore the expression pattern, biological functions and prognostic value of CTSB for gliomas, especially focusing on its role in glioma-related immune responses and immune cells infiltration. So, we chose The Cancer Genome Atlas (TCGA) dataset as training cohort and the Chinese Glioma Genome Atlas (CGGA) dataset as validating cohort to analyze the RNA sequencing (RNA-seq) data of 1024 glioma cases. This study provides a powerful theoretical basis for the design of CTSB-related therapeutics for gliomas.

## Results

### CTSB is remarkably highly-expressed in HGG, isocitrate dehydrogenase (IDH) wildtype, 1p19q non-codeletion type and O^6^-methylguanine-DNA methyltransferase (MGMT) promoter unmethylation gliomas

**Figure 1:**
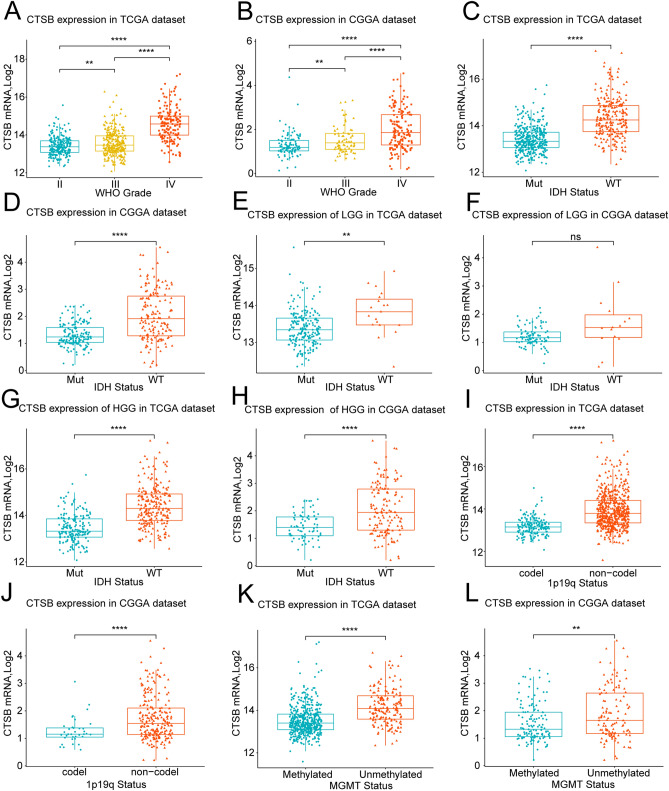
Comparison of the expression of CTSB in gliomas of different WHO grades, isocitrate dehydrogenase (IDH) status, 1p/19q-codeleted status and O^6^-methylguanine-DNA methyltransferase (MGMT) promoter status. CTSB was significantly highly-expressed in high-grade gliomas (HGG), IDH-wildtype, 1p19q non- codeletion and MGMT promoter unmethylation gliomas in TCGA and CGGA datasets. The expression of CTSB in TCGA dataset according to WHO grade (**A**), IDH status (overall WHO grades (**C**), LGG (E), HGG(G)), 1p/19q-codeleted status (**I**) and MGMT status (**K**). The expression of CTSB in CGGA dataset according to WHO grades (**B**) and IDH status (overall WHO grades (**D**), LGG (**F**), HGG(**H**)), 1p/19q-codeleted status (**J**) and MGMT status (**L**). *P < 0.05, **P < 0.01, ***P < 0.001, ****P < 0.0001.

To explore the expression pattern of CTSB in gliomas, we analyzed the RNA-seq data of glioma samples from TCGA and CGGA databases. We found that CTSB was significantly highly-expressed in HGG especially in WHO grade IV gliomas (*P* < 0.0001) (Fig. 1A,B). This result was consistent in both databases. Meanwhile, the different types of IDH status, 1p/19q-codeleted status and MGMT status has been proved to be crucial to clarify the subtypes of gliomas and predict the prognosis of patients^6^. Therefore, we analyzed the difference of the expression level of CTSB between these glioma types. It was showed that the expression of CTSB in IDH wide-type (Fig. 1C,D overall grade; E, F low-grade gliomas (LGG); G, H HGG), 1p19q non-codeletion type (Fig. 1I,J) and MGMT promoter unmethylation type (Fig. 1K,L) were significantly higher than those in IDH mutant-type (especially for HGG), 1p19q codeletion type and MGMT promoter methylation type of gliomas respectively. Among these variables, IDH status was proved to be most strongly associated with CTSB expression via multivariate correlation analysis of TCGA (Fig. S1A) and CGGA (Fig. S1B) cohorts. These results reveal that CTSB is highly-expressed in these malignant molecular types which are negatively markers of therapeutic reactivity and prognosis of gliomas.

### The upregulated CTSB is correlated with the poor prognosis of gliomas

**Figure 2:**
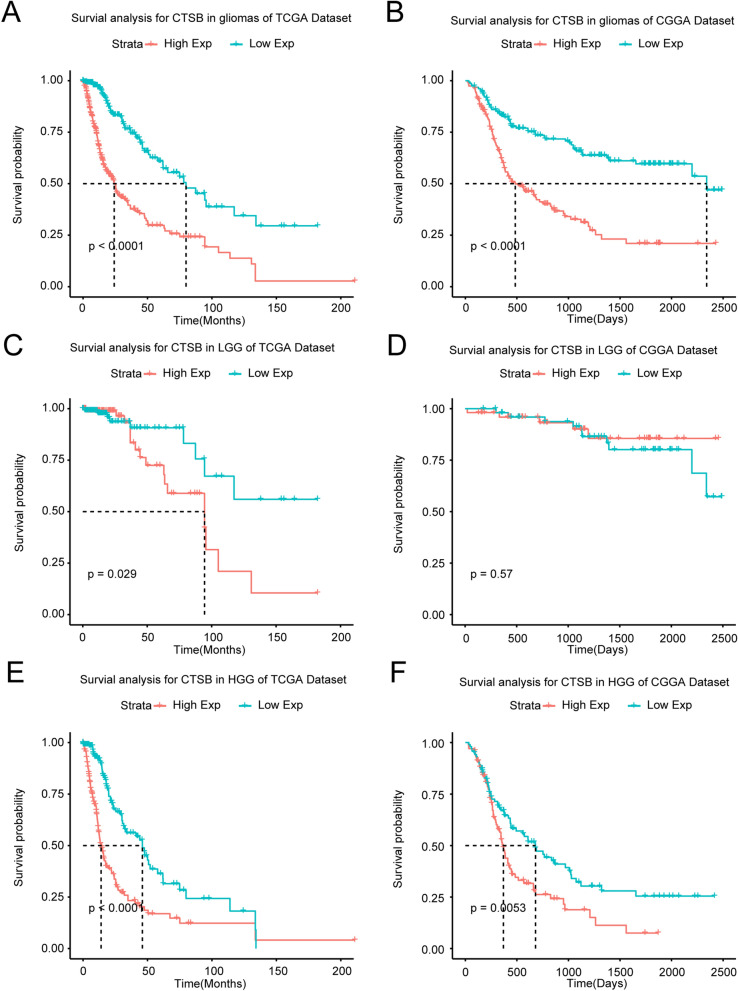
Survival analysis of glioma patients based on CTSB expression in TCGA and CGGA cohorts. Kaplan–Meier analysis indicated that high expression of CTSB was related to significantly worse prognosis overall in overall glioma (**A**,**B**), low-grade gliomas (LGG) (**C**,**D**) and HGG (**E**,**F**) patients.

**Figure 3:**
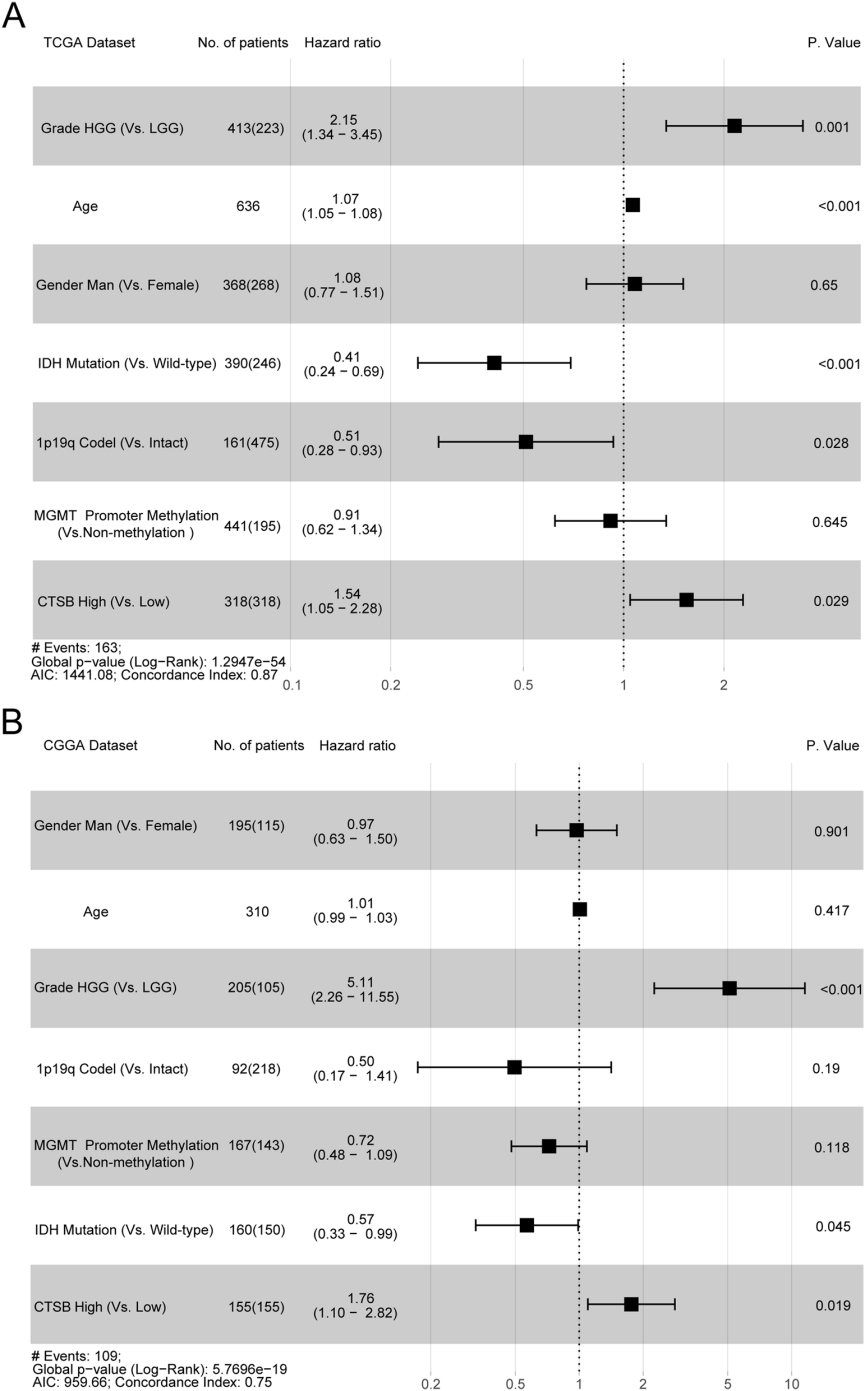
Forest plot of hazard ratios for overall survival rates assessed by CTSB and clinicopathological factors. CTSB was an independent prognostic factor after adjusting for age, WHO grade, IDH status, 1p19q status and MGMT status in TCGA (**A**) and CGGA (**B**) datasets.

The results above suggested that CTSB may be a potential biomarker of malignant gliomas. Then we analyzed the prognostic value of CTSB for gliomas. Firstly, we drew Kaplan Meier curve with the survival data of glioma samples in the two databases (Fig. 2A,B), we found that patients with higher expression of CTSB would have shorter OS time (*P* < 0.0001). To avoid the influence of tumor heterogeneity, we also compared the effect that CTSB have on the OS time of LGG (Fig. 2C,D) and HGG (Fig. 2E,F) respectively, and this phenomenon was more obvious in HGG. Secondly, we further explored the role that CTSB played in the prognosis of gliomas together with other factors such as gender, age, WHO grade, IDH mutant status, MGMT promoter methylation type and 1p19q codeletion status (Fig. 3A,B overall grade; Fig. S2A, B LGG; Fig. S2C, D HGG). The multi-factors COX analysis also come to the same conclusion that high-expressed CTSB is a risk factor and a negative biomarker related with the poor prognosis of HGG.

### CTSB is highly-expressed in mesenchymal subtype of gliomas and is specific and sensitive in predicting mesenchymal subtype

**Figure 4:**
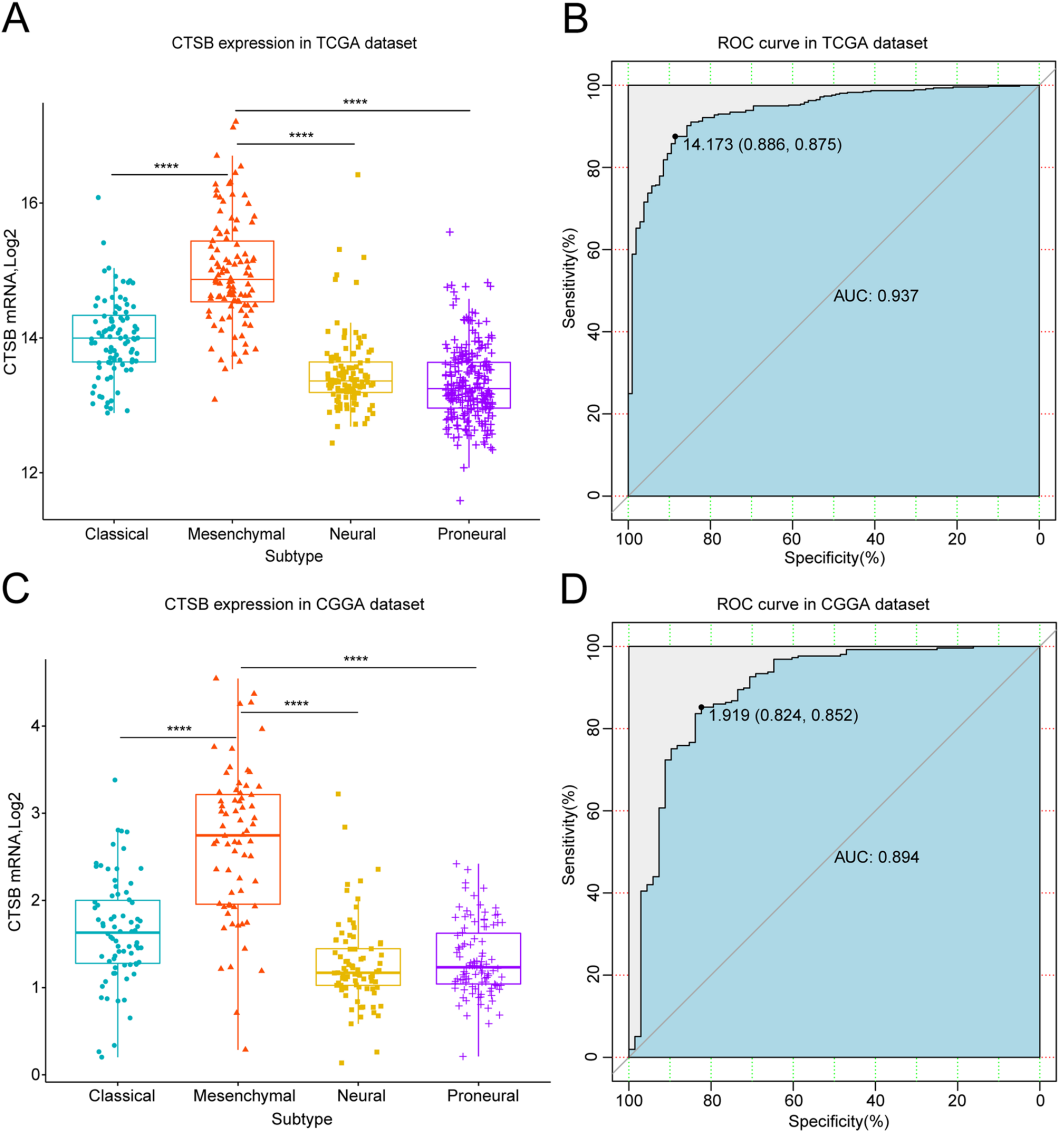
Comparison of CTSB expression levels in different TCGA molecular subtypes. CTSB was significantly enriched in the mesenchymal subtype in TCGA (**A**) and CGGA (**C**) cohorts (*P* < 0.0001). Receiver operator characteristic curve (ROC) curve analysis showed the predictive value of CTSB for mesenchymal subtype in the TCGA and CGGA cohorts (**B**,**D**). *P < 0.05, **P < 0.01, ***P < 0.001, ****P < 0.0001.

In the last few years, TCGA network have divided gliomas into four molecular subtypes: proneural, neural, classical and mesenchymal type^36^. This classification has been proved to be meaningful for patients’ survival status, especially mesenchymal subtype which is representing for invasion and poor prognosis^5^. So, we analyzed the relationship between CTSB and TCGA subtypes. We found that CTSB was remarkably highly-expressed in mesenchymal subtype both in TCGA and CGGA databases (Fig. 4A,C). Then we performed receiver operator characteristic curve (ROC) to evaluate the specificity and sensitivity of CTSB in predicting mesenchymal subtype gliomas. The area under the curve (AUC) of the TCGA dataset was 0.937, and the specificity and sensitivity were 88.6% and 87.5% respectively at the optimal cut-off value of 14.173 (Fig. 4B). The AUC of the CGGA dataset was 0.894, and the specificity and sensitivity were 82.4% and 85.2%, respectively at the optimal cut-off value of 1.919 (Fig. 4D). These results indicate that CTSB is highly specific to the mesenchymal gliomas and can be used to predict this subtype.

### CTSB is closely related to the immune activities in gliomas

**Figure 5:**
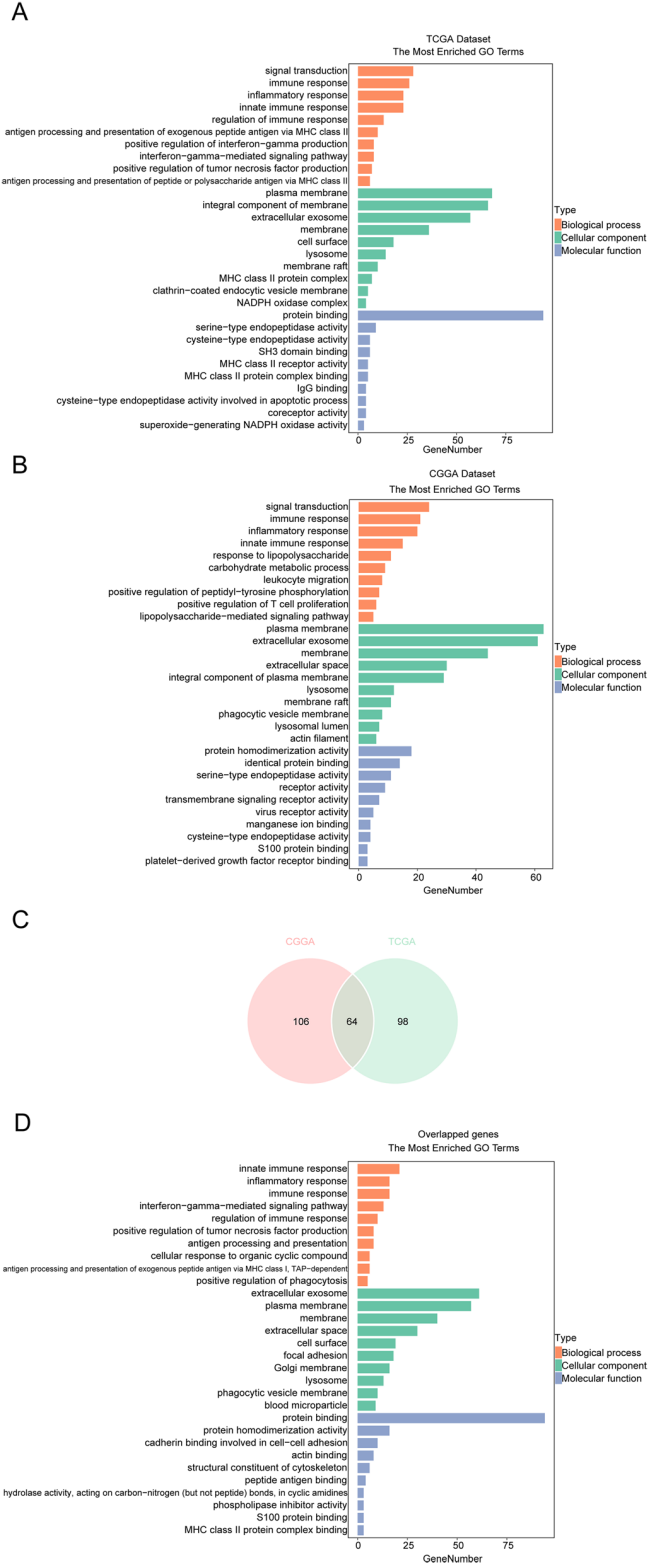
Gene ontology (GO) analysis of CTSB-related characteristics in gliomas. The results revealed that CTSB is related to some important biological processes in glioma based on TCGA (**A**) and CGGA (**B**) datasets. GO analysis of 64 genes common to both datasets was used for validation (**C**,**D**).

To further study the feature and biological function of CTSB, we ranked the genes related with CTSB via Spearman’s correlation analysis, finally we respectively screened 162 related genes from TCGA database (151 positively corelated genes, 11 negatively corelated genes, |R|> 0.7 and *P* < 0.05) and 170 related genes from CGGA database (165 positively corelated genes, 5 negatively corelated genes, |R|> 0.6 and *P* < 0.05), as shown in Supplementary Table S1. Then we used the DAVID website to perform Gene ontology (GO) functional analysis of these genes. The biological processes of CTSB mainly contain signal transduction, immune response, inflammatory response, innate immune response, regulation of immune response, interferon-gamma-mediated signaling pathway, leukocyte migration, positive regulation of T cell proliferation, positive regulation of tumor necrosis factor and interferon-gamma production, antigen processing and presentation, positive regulation of phagocytosis and so on. In terms of cellular components, CTSB mainly act as extracellular exosome, membrane raft, actin filament and lysosome, which located on cell surface, phagocytic vesicle membrane, plasma membrane and in extracellular space. The molecular function of CTSB mainly contains protein binding, serine-type and cysteine-type endopeptidase activity, hydrolase activity, protein homodimerization activity, S100 protein and MHC class II protein complex binding and so on. The above results were consistent among TCGA and CGGA databases (Fig. 5A,B). Meanwhile, we also did GO functional analysis on the overlapped 64 related genes (Supplementary Table S1) of the two databases for validation (Fig. 5C,D). Our study showed that CTSB is correlated with many immune activities and inflammatory response processes in gliomas, and mainly act as extracellular exosome, lysosome and membrane raft in extracellular space or on cell membrane.

**Figure 6:**
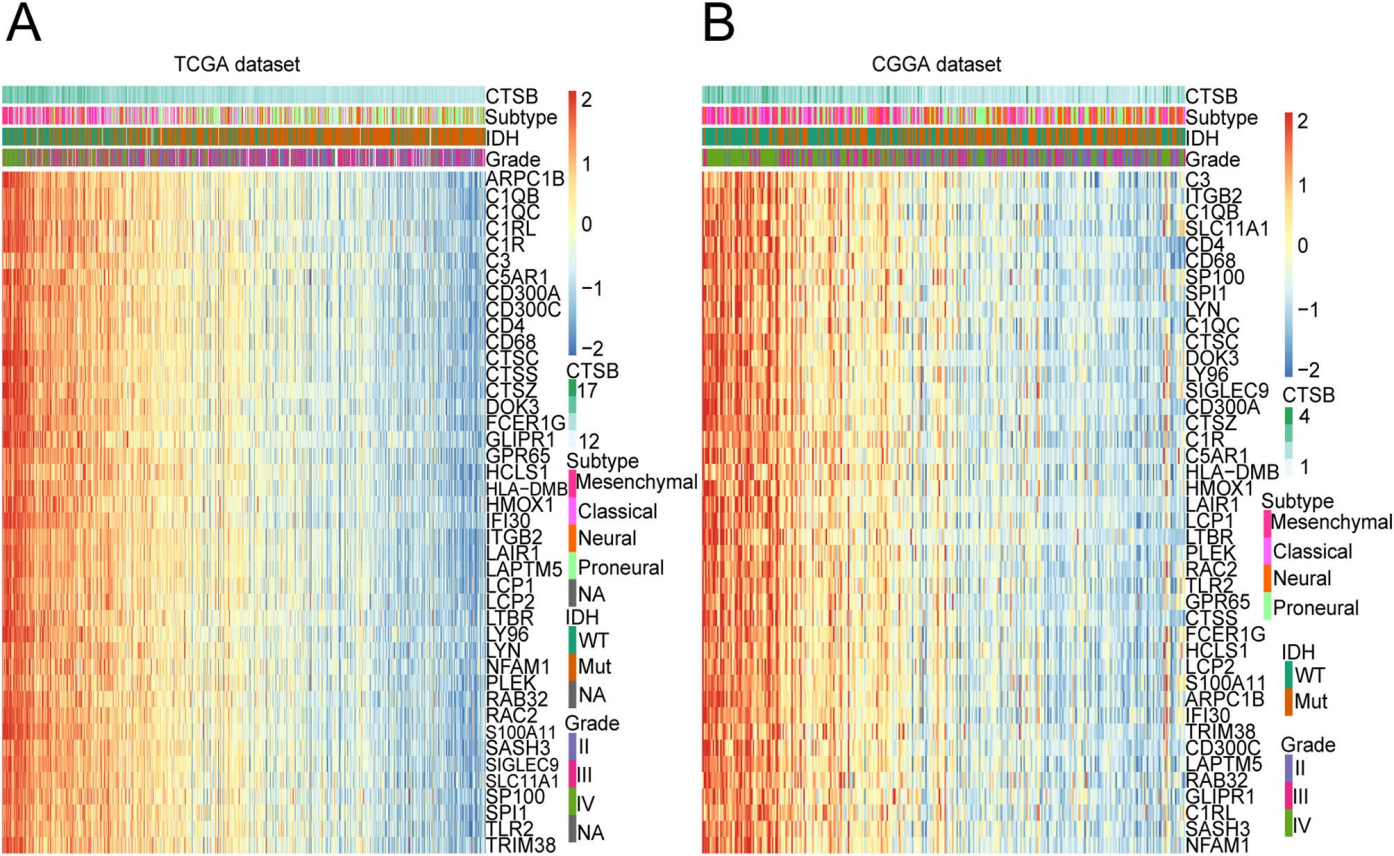
Heatmap analysis of the relationship between CTSB and immune function-related genes in gliomas. The results showed that CTSB had a markedly positive correlation with most immune genes in both TCGA (**A**) and CGGA (**B**) cohorts.

Then for further exploration of the function in glioma-related immune activities, we download the immune genes sets from AmiGO2 website. According to this dataset, we chose 104 genes from TCGA database (|R|> 0.7, *P* < 0.05) and 94 genes from CGGA database (|R|> 0.6, *P* < 0.05) which was significantly correlated with CTSB (Supplementary Table S2). Then the overlapped 42 immune-related genes of them (Supplementary Table S2) were used to draw the heat map (Fig. 6A,B). Then we found that most of these genes are positively related to the expression of CTSB in both databases, which further reveals the role that CTSB plays in glioma-related immune activities.

### CTSB is closely related to the inflammatory response of gliomas

**Figure 7:**
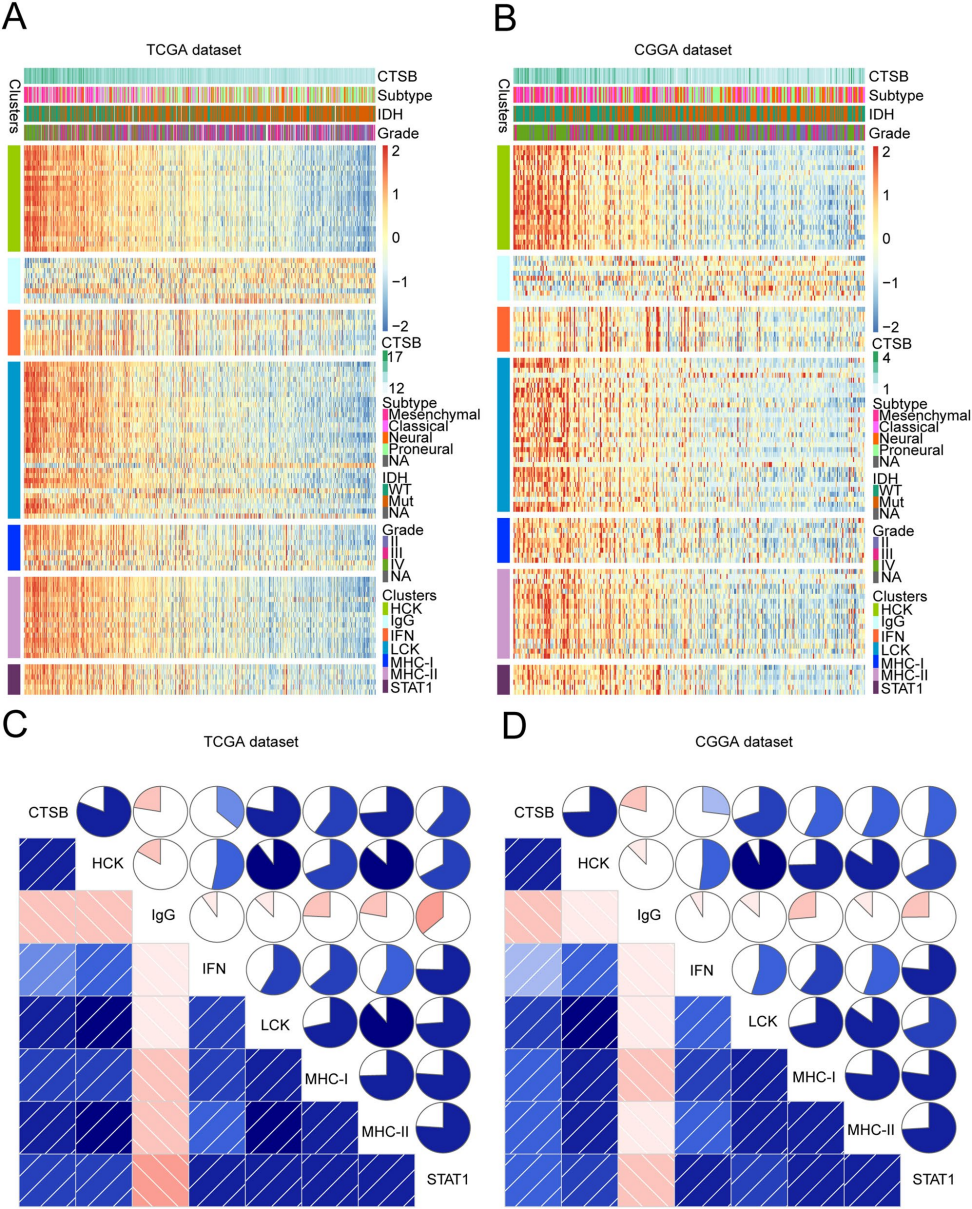
CTSB-related inflammatory activities in gliomas. The relationship between CTSB and WHO grade, IDH status, molecular subtypes and inflammatory metagenes are presented as a heatmap in TCGA and CGGA databases (**A**,**B**). Correlograms showed the correlation between CTSB and inflammatory metagenes (**C**,**D**). Blue represents positive correlations, and red represents negative correlations. Color intensity and the size of the circle in pie charts are proportional to the correlation coefficients. The results indicated that CTSB was significantly positively correlated with most inflammatory activities.

In the results above, it is suggested that CTSB is related with the inflammatory response of gliomas, so in order to explore the function of CTSB, we analyzed 104 inflammatory genes which could be divided into seven metagenes^5^. Supplementary Table S3 summarizes the detailed list of these genes. In TCGA and CGGA datasets, we performed heatmap clustering analysis on the above genes and found that all of the gene metagenes are positively associated with CTSB except IgG (Fig. 7A,B; Fig. S3A, B). To validate this result, we applied gene set variation analysis (GSVA) to draw correlograms according to the Pearson correlation between CTSB and the seven gene metagenes (Fig. 7C,D). Finally, the results of the two cohorts were highly consistent with the heatmap. The expression of CTSB is remarkably positively related with HCK, MHC-II, STAT1, LCK, MHC-I and interferon (IFN) metagenes, but negatively associated with IgG metagene.

### Relationship between CTSB and tumor-infiltrating immune cells in gliomas

**Figure 8:**
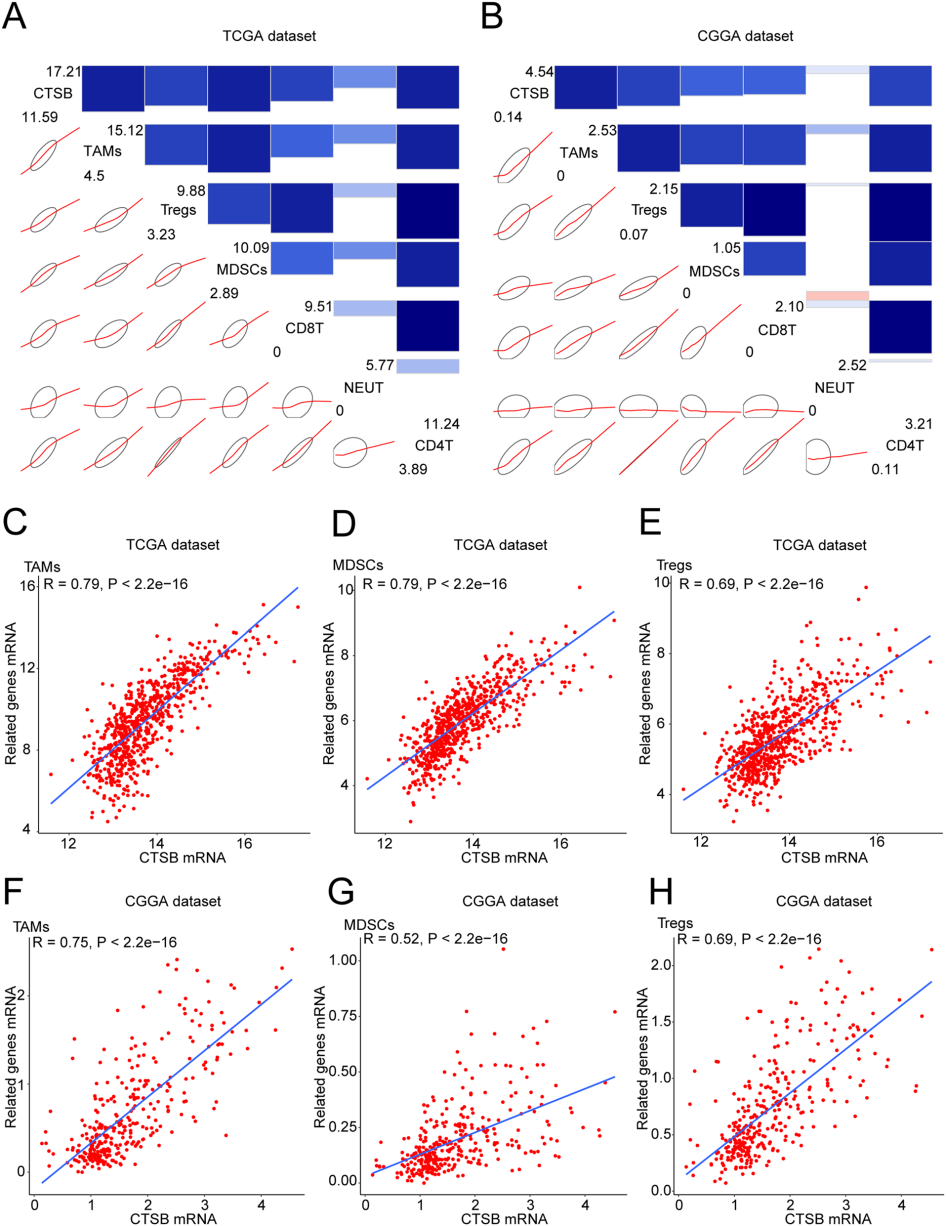
CTSB-related infiltrating immune cells in gliomas. (**A**,**B**) Correlograms showing the correlation between CTSB and immune cell infiltration level based on both datasets. Blue represents positive correlations, and red represents negative correlations. Color intensity and the size of the bars and ellipses are proportional to the correlation coefficients. The leading diagonal contains the minimum and maximum values of variables. (**C**–**H**) CTSB was significantly positively correlated with tumor-related macrophages (TAMS), myeloid-derived suppressor cells (MDSCs) and regulatory T cells (Tregs) in both datasets. Each point represents a glioma sample. A regression line was fitted to the dot plot.

Tumor-infiltrating immune cells were proved to be essential in the invasion progress of gliomas^37^. Therefore, it is necessary to explore the relationship between CTSB and infiltrating immune cells in gliomas. We analyzed the most common 6 types of immune cells such as CD4 + T cells, regulatory T cells (Tregs), CD8 + T cells, tumor-associated macrophages (TAMs), myeloid-derived suppressor cells (MDSCs) and neutrophils (NEUT), and the detailed information of the markers of these 6 cell types is summarized in Supplementary Table S4. Then we drew corrgrams to show the correlation between CTSB and above cell types in the two databases. It was shown that these infiltrating immune cells were positively correlated with the expression of CTSB (Fig. 8A,B). We also performed Pearson correlation analysis, and CTSB was significantly correlated with TAMs, MDSCs and Tregs in both databases: TAMs (r = 0.79 in TCGA dataset, r = 0.75 in CGGA dataset; Fig. 8C,F), MDSCs (r = 0.79 in TCGA dataset, r = 0.52 in CGGA dataset; Fig. 8D,G), and Tregs (r = 0.69 in TCGA and CGGA datasets; Fig. 8E,H).

## Discussion

To date, even lots of trials of targeted therapy for gliomas have not shown anticipated efficacy, enrichment strategies with more precise biomarkers of gliomas will greatly increase the chances of success in the future^4,5^. Our study indicates that highly-expressed CTSB in the immune microenvironment is closely correlated with the malignant molecular features, worse prognosis, immune cells infiltration and immunosuppression processes of gliomas, which can be regard as a new prognostic biomarker and potential therapeutic target.

First, we found that CTSB is significantly highly-expressed with increasing WHO grade, especially for HGG, which was consistent with previous studies^28^. We also demonstrated that CTSB is highly-expressed in known malignant glioma molecular phenotypes such as IDH wild-type, MGMT promoter unmethylation, 1p19q non-codeletion and mesenchymal subtype. Meanwhile, CTSB may be a specific and sensitive predictor of mesenchymal subtype gliomas. According to the clinical meaning of these molecular phenotypes in gliomas, patients with highly-expressed CTSB may have a greater risk of tumor recurrence, progression, epithelial mesenchymal transformation (EMT) and therapeutic resistance. The survival analysis showed that the highly-expressed CTSB means shorter OS time of glioma patients, which is also an independent risk factor for the prognosis, and the similar result was reported before in GBM^38^. The overall expression and enzymatic activity of CTSB in GBM could be regulated through the alternative splicing of the pre-mRNA of CTSB^39,40^, which determined by the differentiation and microenvironment of tumor cells and in return influence the malignant behavior of GBM^39^. We preliminarily profiled the splicing patterns of CTSB for gliomas via TCGA SpliceSeq^41^, and the exon 8 skip is the most common splicing type of CTSB in gliomas (Fig. S4A-E). CTSB also acts as a mediator of EMT process upregulating EMT-activated transcription factors through the Wnt/β-catenin pathway^15^. As the most invasive lysosomal cathepsin in GBM, CTSB remodels ECM to create prerequisite niches for invasion^33^ by degrading ECM components(such as laminin, collagen, fibrin, tenascin-C)^42^, triggering the uPA/plasminogen/plasmin proteolytic cascade^42^, activating other proteolytic enzyme systems^32^, releasing cytokines and growth factors, aggravating peritumoral edema and acidic microenvironment^43^. It also impairs the basement membrane of blood brain barrier (BBB) to promote glioma cells invasion^33^. CTSB also induces cell senescence mediated malignant transformation via CCNB2/SASP/CTSB & PGE2 axis^44^. It was also shown that CTSB can promote proliferation by upregulating the expression of p-ERK and c-Myc and reducing the level of Cellular Repressor of E1A Stimulated Genes 1 in GBM^45^. CTSB can increases the resistance of glioma cells to apoptosis and ferroptosis via lysosome-nuclear pathway of communication^15,46^. Highly-expressed CTSB interacts with the ANXA2 tetramer and induces the expression of VEGF-C, TGF-β and matrix metalloprotein (MMP)-9 to promote high permeability angiogenesis in gliomas^8,47^. CTSB promotes the generation and proliferation of GSCs via renin-angiotensin system (RAS)^28^, and regulates the self-renewal of GSCs through hedgehog components, Bmi1 and Sox2^29^. These studies strongly support our conclusion. Therefore, CTSB can be used for the molecular pathology diagnosis and prognosis evaluation of gliomas as a promising biomarker.

Regarding the role and biological processes of CTSB in gliomas, we found that CTSB is mainly related to signal transduction, immune and inflammatory response, regulation of immune response, leukocyte migration, interferon-gamma-mediated signaling pathway, antigen processing and presentation. It also associates with the positive regulation of T cell proliferation, tumor necrosis factor and interferon-gamma production. Correlation analysis confirmed that glioma-associated immune response and inflammatory activity are significantly correlated with CTSB. Immune cell infiltration and immunosuppression play a vital role in the progression and treatment resistance of gliomas^3,5^. But the exact function of CTSB in these processes remains unclear. Our data showed that the degree of immune cell infiltration is significantly related to the expression level of CTSB, especially the immunosuppression-related immune cells TAMs, MDSCs and Tregs. This new finding suggests that CTSB in TME may associate with the immunosuppression and progression of gliomas. Previous studies reported that CTSB is actively correlated with T lymphocytes apoptosis in antigenic immune response^48^ and delayed type hypersensitivity as a regular factor of lysosomal biogenesis and autophagy^49^. It induces cognitive impairment through inflammatory response and drives inflammatory brain disease via regulating the production and secretion of IL-1β^21^. CTSB can activate NLRP3 inflammasome to adjust IL-1β and IL-18 production^7^, and it also controls the secretion of TNF-α in monocytes and IL-12 in macrophages^50^. It may cause anti-inflammatory response via adjusting autophagy or mitochondrial dynamics of macrophages^51^. CTSB is highly-expressed in lung cancer and recruits monocytes into tumor to become TAMs, which promotes immunosuppression and tumor progression^52^. CTSB knock-down significantly decreases MDSCs infiltration of premalignant intestinal polyps^53^. CTSB-mediated CD18 flaking adjusts the extravasation, transmigration and recruitment of leukocyte from tumor angiogenic vessels^47^. CTSB is related to infiltrating immune cells with the activation of ELR chemokines and inactivation of Non-ELR chemokines^54^. CTSB cracks Rip1 kinase to inhibit the necrotic apoptosis of TAMs and promotes macrophage-assisted pro-metastatic processes^55^. CTSB in TAMs creates an advantageous microenvironment for tumor invasion^19^ and protects tumor cells from apoptosis induced by etoposide, paclitaxel and doxorubicin^56^. As proved in mouse model, CTSB in MDSCs is crucial for the tumorigenesis of pancreatic neuroendocrine carcinoma^57^. Interplay between CTSB and NLRP3 inflammasome in tumor-infiltrating MDSCs results in IL-1 β production and pro-tumor immune response^58^. CTSB also participates in antigen-presenting, immune cells differentiation and homeostasis^48^. CTSB is closely related to immunosuppression of cervical cancer^59^. The CTSB on tumor cells surface can remove the autoreactive lymphocytes and degrade these cytotoxic effector molecules (such as IgG and chemokines CXCR3, CXCL9, CXCL10) synthesized from tumor-suppressive infiltrating immune cells^7,60^. CTSB promotes the apoptosis of CD8 + T lymphocytes to prevent them from being memory CD8 + T cells and decrease the lasting maintenance of them^48^. It also induces the programmed death of pro-B cells mediated with CpG TLR-9 and the death of B cells from antigen-dependent germinal center^61^. Therefore, the role of CTSB in glioma-related immune response and immunosuppression makes it possible to use CTSB as a therapeutic target enhancing the host immune system to interfere with immune evasion of glioma cells, which is a promising prerequisite in improving the reactivity of targeted therapy for gliomas^62^.

According to the expression patterns, prognostic significance and the biological processes of CTSB in gliomas, we deem that CTSB-based therapy might be an important strategy to improve the overall prognosis and the therapeutic reactivity of glioma patients. BBB-permeable CTSB inhibitors have been used in many neurological diseases such as stroke, Alzheimer’s disease and Parkinson’s disease^21^. Recently, many researchers explored the therapeutic application of small molecule inhibitors for CTSB in many cancers^7^, and CTSB-based visible-light-triggered nanoparticles of prodrug can enhance the effect of immune checkpoint blocking therapy, chemotherapy and photodynamic therapy^63^. Moreover, previous studies have preliminarily investigated the potential clinical value of CTSB for gliomas in vitro and vivo^8,28,31,64^. Down-regulation of CTSB significantly inhibited the proliferation, invasion and tumor angiogenesis of GBM^8^. Auranofin and Tivozanib can reduce the invasion of GBM cells through inhibiting the activity of CTSB and blocking the CTSB/uPA/MMP-2 proteolytic cascade respectively^31,64^. Considering the interaction between CBST and RAS in promoting GSCs invasion, targeted therapy combined CTSB with RAS remarkably reduces the growth of GBM in vivo^28^. For treatment resistance, overexpressed CTSB promoted EMT, decreased the cytotoxicity of TMZ and reduced TMZ-induced cell death in GBM^35^. High expression of CTSB also facilitates radioresistance of GBM and paediatric glioma via increasing homology recombination^65^. Inhibition of CTSB will upregulate the expression of γH2AX and H2AX by inhibiting C-Met signal transduction, resulting in the transcriptional arrest of cells that makes them sensitive to apoptosis induced by radiation, thus improving the radioresistance of gliomas^66^. Furthermore, the specific expression of CTSB in GBM has been attempted to apply in the design of surgical protocols^67^. CTSB-based small molecular probes of suicide inhibitors with fluorescently quenched activity have been demonstrated in mouse models that they can quickly recognize the core and edge of GBM, rapidly visualizing marginal infiltrating tumor cells during operation, which provides an innovative imaging method of potential application value for surgical directional resection of gliomas^67^. Based on our findings and the above studies, CTSB-targeted therapy alone or combined with immunotherapy and vascular targeting therapies may complement current treatment strategies for gliomas. Nevertheless, further studies with single-cell RNA sequencing are currently in progress to optimize the limitations of multicellular level analysis for TCGA and CGGA datasets, which can also be used to furtherly explore splice variant expression and other regulation ways to reveal the specific mechanism of CTSB in the immunosuppressive microenvironment of gliomas. More evidences of preclinical studies are also needed to support the clinical application of CTSB. In the future, CTSB may clinically acts as a potential biomarker and practical target for the precision diagnosis, treatment, image-guided surgery, cellular modification agent and targeted drug delivery of gliomas^17^.

In brief, this research mainly focused on the expression pattern, biological function and clinical significance of CTSB in gliomas. Our study suggested that highly-expressed CTSB in gliomas was closely associated with the malignant pathological subtypes, poor prognosis, tumor-infiltrating immune cells and immunosuppression, which made it a promising biomarker and potential target in the diagnosis, treatment and prognostic assessment of gliomas. We believe that in the future, CTSB-based therapies alone or in combination with other treatments will be a meaningful strategy in the combination and individualized precision therapies for gliomas.

## Methods

### Patients and samples

All the selected cases of glioma ranging from WHO grade II to grade IV were collected from the TCGA database (699 cases) (http://cancergenome.nih.gov/) and the CGGA database (325 cases) (http://www.cgga.org.cn). The CGGA database was mainly used to avoid the limitation of single-dataset research. We obtained all the data of RNA-seq, molecular pathological materials and the overall survival (OS) rate information from above cases. We sifted through and removed 63 cases and 15 cases without available above materials from the TCGA and CGGA datasets respectively. This research was approved by the Ethics Committee of the Peking University Third Hospital (S2020018).

### Statistical analysis

We performed all the statistical analyses and figures with R software for MacOS, version 4.0.3 (http://www.r-project.org). All the gene expression profiling data in this study were log-transformed for further analysis. Kaplan–Meier survival curves were used to show the overall survival rate difference of glioma patients. Multivariate Cox regression analysis was performed with the survival package in R. Other R packages such as ggplot2, pROC, pheatmap, devtools, corrplot, ggpubr and corrgram package were also used to visualize the analyzed data. We filtered out these genes which were significantly corelated with CTSB via Spearman’s correlation analysis. GO functional analysis was performed using DAVID Bioinformatics Resources 6.8 (https://david.ncifcrf.gov/) to explore the enriched biological process, molecular function and cellular component. The immune system gene sets were downloaded from the AmiGO 2 website (http://amigo.geneontology.org/amigo) to analyze the role that CTSB plays in glioma-related immune activities. The Pearson’s correlation analysis was also used to evaluate the strength of correlation. One-way ANOVA was used to test the differences among at least 3 groups. Student’s t-test was used to test the differences of each 2-group comparison. The statistically significant differences were defined at the level of *P* < 0.05.

### Ethics approval

This research was approved by the Ethics Committee of the Peking University Third Hospital (S2020018).

## Supplementary Information

Supplementary Information 1.

Supplementary Information 2.

Supplementary Information 3.

Supplementary Information 4.

Supplementary Information 5.

Supplementary Information 6.

Supplementary Information 7.

Supplementary Information 8.

## Data Availability

The datasets presented in this study can be found in online repositories. The names of the repository/repositories and accession number(s) can be found in the article/ Supplementary Material.

## Abbreviations

CNS: Central nervous system
TTF: Tumor treating fields
HGG: High-grade glioma
CTSB: Cathepsin B
ANXA2: Annexin A2
ECM: Extracellular matrix
GBM: Glioblastoma
GSCs: Glioblastoma stem cells
TCGA: The Cancer Genome Atlas
CGGA: Chinese Glioma Genome Atlas
RNA-seq: RNA sequencing
IDH: Isocitrate dehydrogenase
MGMT: O^6^-methylguanine-DNA methyltransferase
LGG: Low-grade glioma
ROC: Receiver operating characteristic
AUC: Area under the curve
GO: Gene ontology
GSVA: Gene set variation analysis
IFN: Interferon
Tregs: Regulatory T cells
TAMs: Tumor-associated macrophages
MDSCs: Myeloid-derived suppressor cells
NEUT: Neutrophils
EMT: Epithelial mesenchymal transformation
BBB: Blood brain barrier
MMP: Matrix metalloprotein
RAS: Renin-angiotensin system
OS: Overall survival

## Code availability

Custom code used to analyse the RNA-seq data and datasets generated and/or analysed in the current study are available from the corresponding authors upon request.
