# Supplementary figures and images for: CTSB is a negative prognostic biomarker and therapeutic target associated with immune cells infiltration and immunosuppression in gliomas
Source: Sci Rep. 2022 Mar 11;12:4295. doi: 10.1038/s41598-022-08346-2 (PMC8917123; doi:10.1038/s41598-022-08346-2)

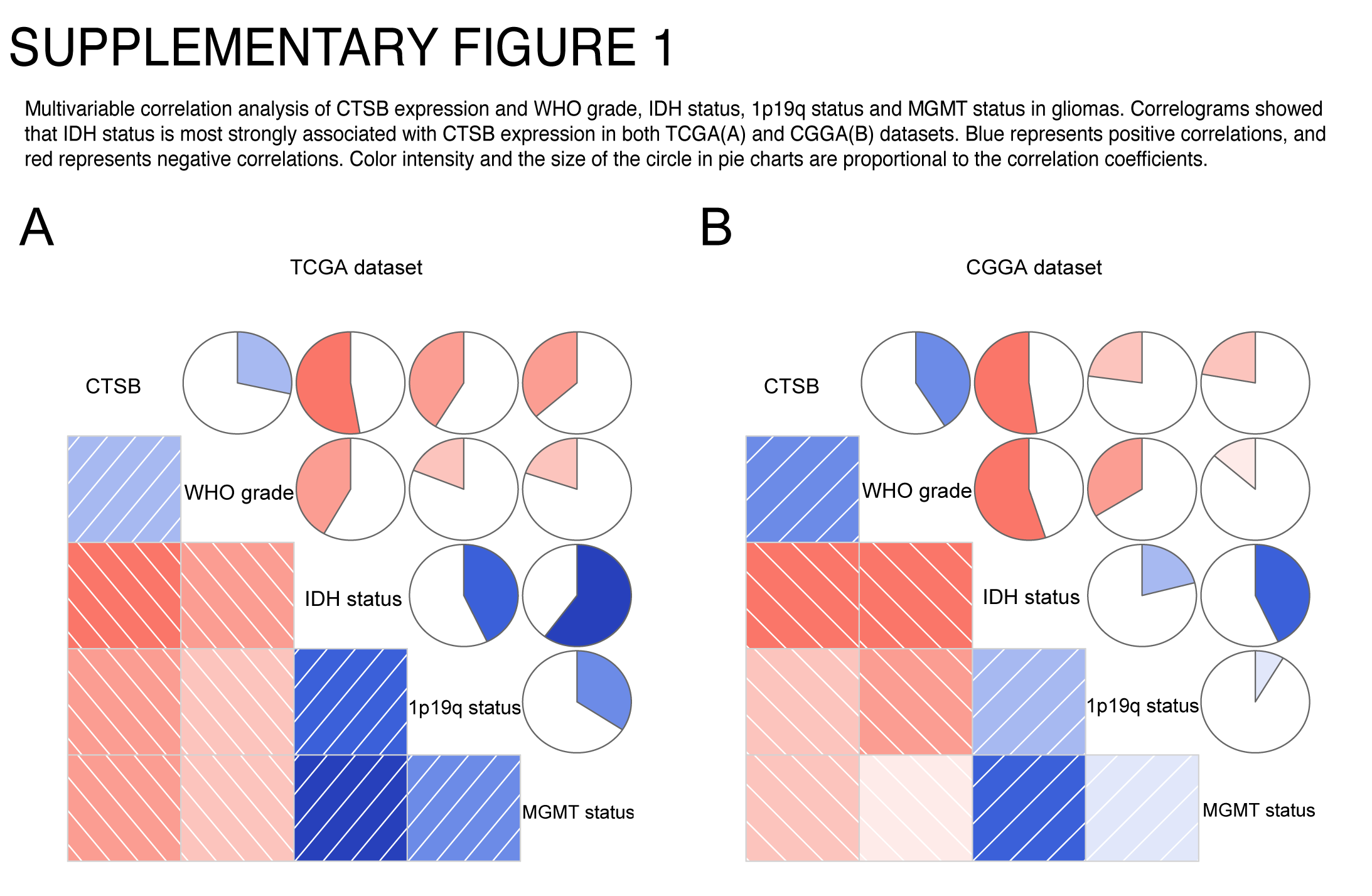

Supplement: Supplementary file 1 — Supplementary Information 1. [file 41598_2022_8346_MOESM1_ESM.tif]

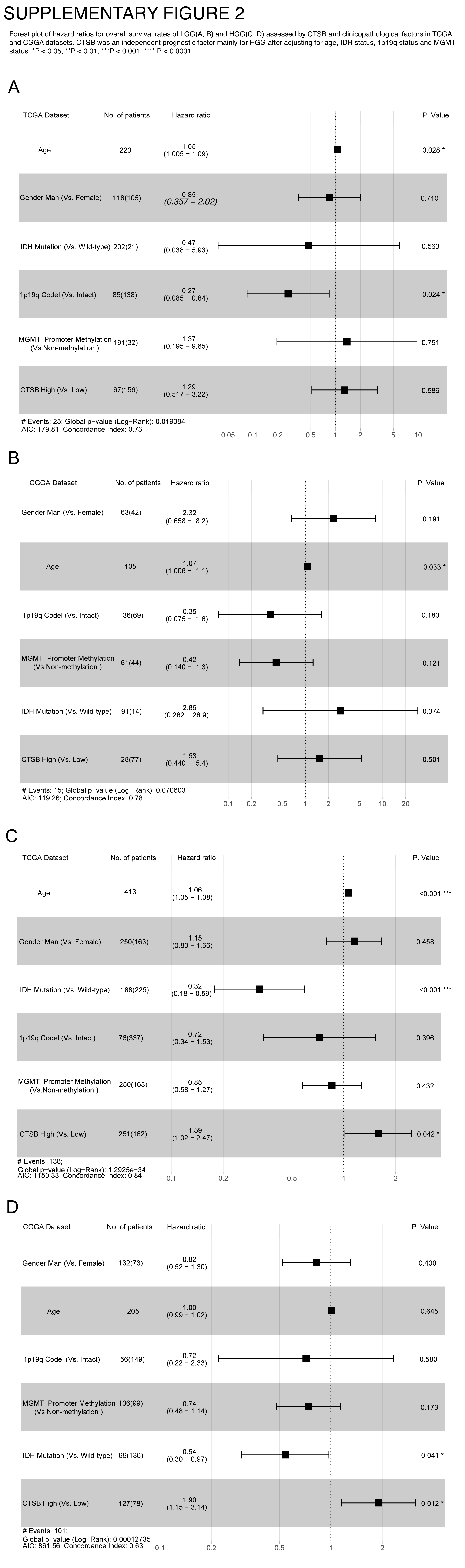

Supplement: Supplementary file 2 — Supplementary Information 2. [file 41598_2022_8346_MOESM2_ESM.tif]

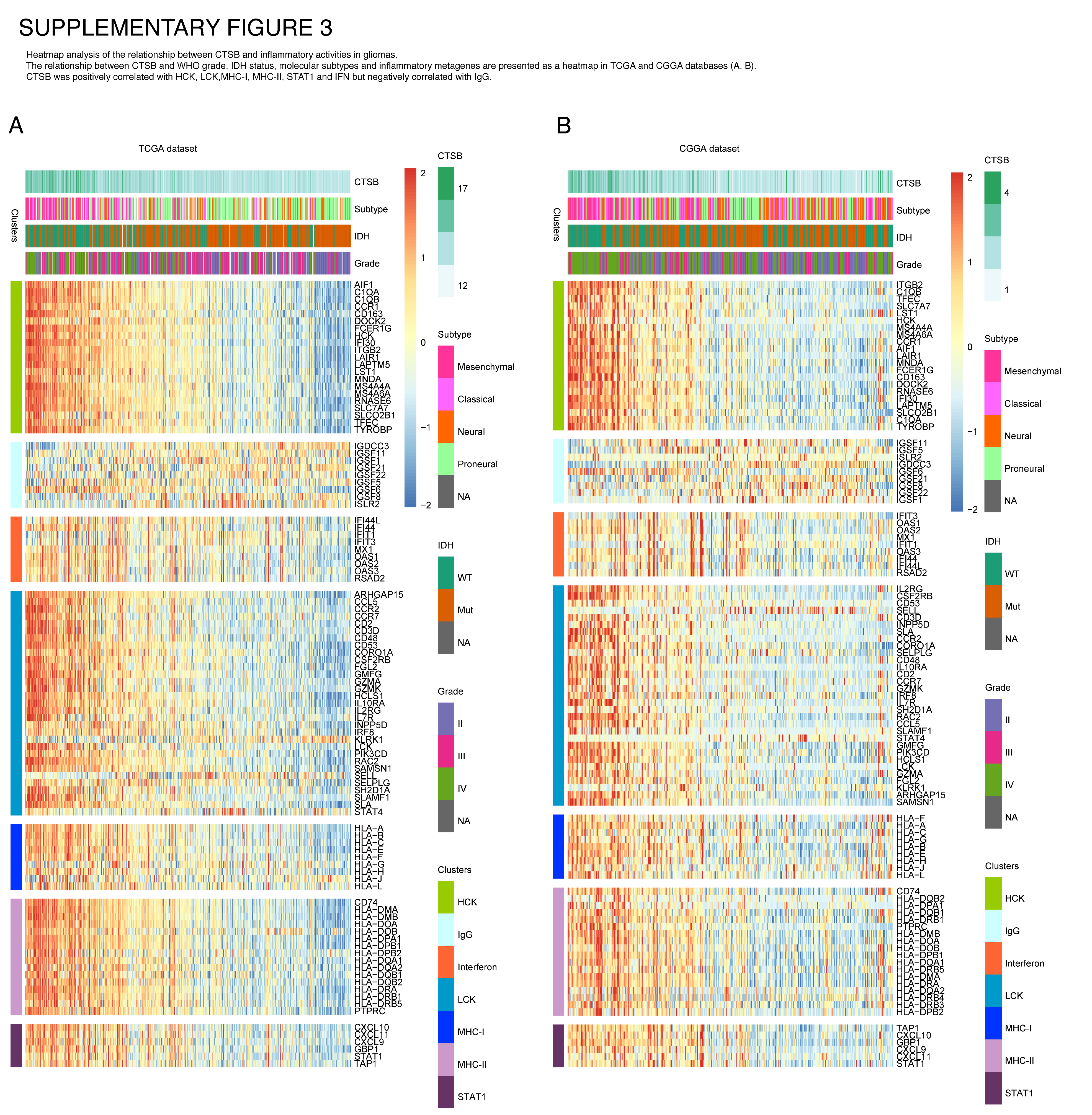

Supplement: Supplementary file 3 — Supplementary Information 3. [file 41598_2022_8346_MOESM3_ESM.tif]

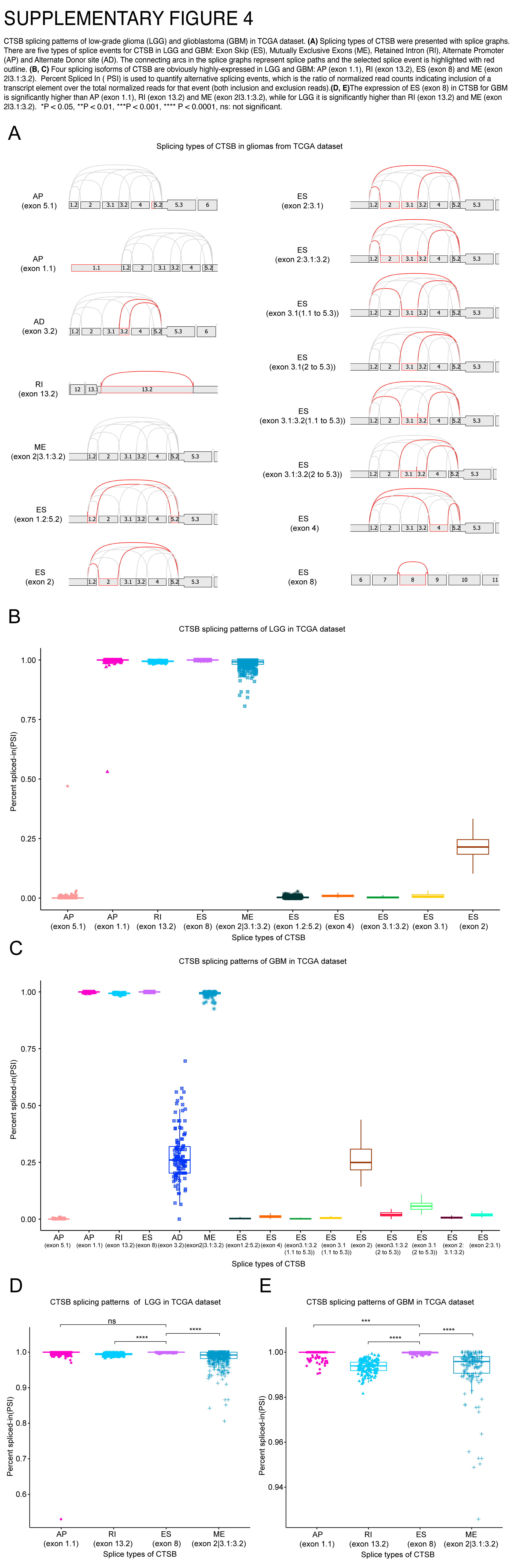

Supplement: Supplementary file 4 — Supplementary Information 4. [file 41598_2022_8346_MOESM4_ESM.tif]
