# Supplementary material for: CTSB is a negative prognostic biomarker and therapeutic target associated with immune cells infiltration and immunosuppression in gliomas
Source: Sci Rep. 2022 Mar 11;12:4295. doi: 10.1038/s41598-022-08346-2 (PMC8917123; doi:10.1038/s41598-022-08346-2)
Supplement: Supplementary file 5 — Supplementary Information 5. [file 41598_2022_8346_MOESM5_ESM.pdf]

Supplementary Table S1. Genes associated with CTSB in TCGA and CGGA datasets.
There are 162 related genes from TCGA database (151 positively corelated genes, 11 negatively corelated genes) and 170 related genes from CGGA database (165 positively corelated genes, 5 negatively corelated genes). The overlapped 64 CTSB-related genes of TCGA and CGGA datasets are highlighted with red color.

TCGA Dataset

Genes	R
LRRC16B	−0.8
TTC3	−0.8
ZNF711	−0.8
LRRC37B	−0.8
ZDHHC22	−0.8
SS18L1	−0.8
CDHR1	−0.8
ACVR2B	−0.8
EHMT2	−0.8
BCL7A	−0.8
ZNF638	−0.8
CASP8	0.8
CAP1	0.8
HLA−DPB1	0.8
ADORA3	0.8
PQLC3	0.8
TBXAS1	0.8
GPX1	0.8
RIPK3	0.8
SLA	0.8
CLEC7A	0.8
HCST	0.8
SAT1	0.8
PLEK	0.8
NCF4	0.8
VSIG4	0.8
PLA2G15	0.8
TRIM38	0.8
NFAM1	0.8
GNA15	0.8
TREM2	0.8
NUDT16P1	0.8
TYMP	0.8
HLA−DPA1	0.8
TMEM109	0.8
RNASET2	0.8
PTGS1	0.8
HLA−DOA	0.8
C17orf87	0.8
FXYD5	0.8
ADPRH	0.8
CYTH4	0.8
LCP2	0.8
PTPN6	0.8
ELF4	0.8
SP100	0.8
LAT2	0.8
ABI3	0.8
C1RL	0.8
C5AR1	0.8

CGGA Dataset

Genes	R
CRHR1−IT1	−0.7
RUNDC3A	−0.7
HNRNPUL2	−0.7
MCMDC2	−0.7
PDZD4	−0.7
PRSS23	0.7
EMP1	0.7
IFNGR2	0.7
C1RL	0.7
ARHGAP18	0.7
EMB	0.7
MAPKAPK2	0.7
PLXND1	0.7
M6PR	0.7
C1QB	0.7
SLC1A5	0.7
SP100	0.7
SRPX2	0.7
RDH10	0.7
PGM2	0.7
ANXA2	0.7
PLK3	0.7
ITGA5	0.7
GUSB	0.7
CORO1A	0.7
ARHGDIB	0.7
ITGB3	0.7
GPRIN3	0.7
TPM4	0.7
LRP10	0.7
FOSL2	0.7
HLA−DMB	0.7
ICAM1	0.7
SCPEP1	0.7
WIPI1	0.7
EVI2B	0.7
HCLS1	0.7
OSMR	0.7
FCGRT	0.7
IRAK1	0.7
SLC17A9	0.7
GNA15	0.7
TRIM38	0.7
SIGLEC9	0.7
CYTH4	0.7
SLAMF8	0.7
TMEM154	0.7
SSR3	0.7
AIM1	0.7
WDR1	0.7

CASP1	0.8	RELB	0.7
PLAUR	0.8	SRPR	0.7
IL18	0.8	GCLM	0.7
IL13RA1	0.8	S100A11	0.7
FCGR1A	0.8	RAB32	0.7
RHOH	0.8	TGFB1	0.7
ARHGAP9	0.8	C3	0.7
GLIPR1	0.8	CD276	0.7
HCK	0.8	BAK1	0.7
RGS19	0.8	ST14	0.7
PYCARD	0.8	LTBP2	0.7
MGST2	0.8	ADPGK	0.7
MS4A6A	0.8	TOR4A	0.7
C1R	0.8	C1QC	0.7
DOK3	0.8	STAT3	0.7
SPP1	0.8	CYBB	0.7
CD33	0.8	GLIPR1	0.7
CLIC1	0.8	SAT1	0.7
CCR5	0.8	EDEM2	0.7
LCP1	0.8	HSD3B7	0.7
UNC93B1	0.8	C5AR1	0.7
TLR2	0.8	HMOX1	0.7
APOBEC3G	0.8	FAM114A1	0.7
ADAP2	0.8	EHD4	0.7
TNFRSF1A	0.8	GALNT2	0.7
CD4	0.8	ST8SIA4	0.7
C3	0.8	SQSTM1	0.7
C16orf54	0.8	CTSL1	0.7
CD14	0.8	ADPRH	0.7
TRPV2	0.8	MVP	0.7
C2	0.8	EHBP1L1	0.7
LHFPL2	0.8	TRPV2	0.7
TMSL3	0.8	TNFSF8	0.7
SLC11A1	0.8	DOCK2	0.7
RNASE6	0.8	CCR1	0.7
OLFML3	0.8	RNF19B	0.7
HMOX1	0.8	TNFRSF1A	0.7
NCF2	0.8	IFI30	0.7
RAB32	0.8	NAGA	0.7
HCLS1	0.8	SPI1	0.7
FPR1	0.8	SRGN	0.7
CYP2S1	0.8	RCAN1	0.7
CD86	0.8	LY96	0.7
LY96	0.8	TLR2	0.7
GBGT1	0.8	TMEM109	0.7
SPINT1	0.8	GPR65	0.7
GLRX	0.8	LRRC25	0.7
CYTIP	0.8	PLEK	0.7
CDCP1	0.8	IQGAP1	0.7
CASP4	0.8	MYO1F	0.7
SCPEP1	0.8	GRN	0.7
CD300C	0.8	NAMPT	0.7
RPS6KA1	0.8	LAP3	0.7

CMTM7	0.8
CYBA	0.8
LYN	0.8
GPSM3	0.8
C1QA	0.8
FCGR3A	0.8
OSCAR	0.8
PLBD1	0.8
HAVCR2	0.8
SPI1	0.8
HLA−DMA	0.8
LTBR	0.8
CD74	0.8
HLA−DRA	0.8
IFI30	0.8
KCNE3	0.8
NCF1	0.8
CAPG	0.8
SLC16A3	0.8
ARL11	0.8
GMFG	0.8
ALOX5	0.8
FERMT3	0.8
GPR65	0.8
RBM47	0.8
NAGA	0.8
FTL	0.8
C1QC	0.8
CTSS	0.8
C1QB	0.8
SASH3	0.8
NPC2	0.8
FBP1	0.8
CD300A	0.8
RAC2	0.8
ITGB2	0.8
CD53	0.8
MSR1	0.8
SERPINB1	0.8
CTSC	0.8
FUCA1	0.8
SIGLEC9	0.8
ARPC1B	0.8
LRRC25	0.8
HLA−DMB	0.8
TYROBP	0.8
LAPTM5	0.8
VAMP8	0.8
ALOX5AP	0.8
SERPINA1	0.8
LAIR1	0.8
CD68	0.8
SLC7A7	0.8

FCER1G	0.7
HK3	0.7
CTSC	0.7
CTBS	0.7
CYTIP	0.7
TRAM1	0.7
SLC11A1	0.7
MYO1G	0.7
A2M	0.7
C1R	0.7
PIK3AP1	0.7
ARL11	0.7
SASH3	0.7
CTSZ	0.7
PTGER4	0.7
CHPF2	0.7
HPS3	0.7
PDCD1LG2	0.7
SLC15A3	0.7
TNFRSF1B	0.7
SH2B3	0.7
MAN2B1	0.7
CSF2RB	0.7
THEMIS2	0.7
DPYD	0.7
RAC2	0.7
B4GALT1	0.7
LYN	0.7
PLA2G15	0.7
TMEM106A	0.7
SOD2	0.7
DOK3	0.7
FAM129A	0.7
CD300C	0.7
OLFML3	0.7
APOBEC3C	0.7
CTSD	0.7
ADAP2	0.7
LCP2	0.7
LCP1	0.7
GNS	0.7
HEXB	0.7
PLBD1	0.7
CDCP1	0.7
CTSS	0.7
TCIRG1	0.7
SEC24D	0.7
NFAM1	0.7
MOB1A	0.7
ARPC1B	0.7
MSR1	0.7
SPATS2L	0.7
C1S	0.7

S100A11	0.8
ARHGDIB	0.8
SQRDL	0.8
FCGR2A	0.8
CTSZ	0.8
FCER1G	0.8

LAIR1	0.7
FERMT3	0.7
TLR1	0.7
CD300A	0.8
LTBR	0.8
ITGB2	0.8
LAPTM5	0.8
SPP1	0.8
ELK3	0.8
PYGL	0.8
MGAT1	0.8
CD4	0.8
CD68	0.8
LHFPL2	0.8
