# Supplementary material for: CTSB is a negative prognostic biomarker and therapeutic target associated with immune cells infiltration and immunosuppression in gliomas
Source: Sci Rep. 2022 Mar 11;12:4295. doi: 10.1038/s41598-022-08346-2 (PMC8917123; doi:10.1038/s41598-022-08346-2)
Supplement: Supplementary file 6 — Supplementary Information 6. [file 41598_2022_8346_MOESM6_ESM.pdf]

**Supplementary Table S2. Immune-related genes significantly related to CTSB in TCGA and CGGA datasets.**

There are 104 immune-related genes in the TCGA database and 94 immune-related genes in the CGGA database which was significantly corelated with CTSB. The overlapped 42 immune-related genes significantly related to CTSB of TCGA and CGGA datasets are highlighted with red color.

| TCGA Dataset | CGGA Dataset |
|---|---|
| Genes | Genes |
| CASP8 | IFNGR2 |
| CAP1 | C1RL |
| HLA−DPB1 | C1QB |
| GPX1 | SP100 |
| RIPK3 | PGM2 |
| SLA | ANXA2 |
| CLEC7A | ITGA5 |
| HCST | GUSB |
| PLEK | CORO1A |
| NCF4 | ITGB3 |
| VSIG4 | HLA−DMB |
| TRIM38 | ICAM1 |
| NFAM1 | EVI2B |
| TREM2 | HCLS1 |
| HLA−DPA1 | OSMR |
| RNASET2 | FCGRT |
| HLA−DOA | IRAK1 |
| LCP2 | TRIM38 |
| PTPN6 | SIGLEC9 |
| ELF4 | SLAMF8 |
| SP100 | WDR1 |
| LAT2 | RELB |
| C1RL | S100A11 |
| C5AR1 | RAB32 |
| CASP1 | TGFB1 |
| PLAUR | C3 |
| IL18 | CD276 |
| IL13RA1 | BAK1 |
| FCGR1A | C1QC |
| RHOH | STAT3 |
| ARHGAP9 | CYBB |
| GLIPR1 | GLIPR1 |
| HCK | HSD3B7 |
| PYCARD | C5AR1 |
| C1R | HMOX1 |
| DOK3 | SQSTM1 |
| CD33 | MVP |
| CCR5 | TNFSF8 |
| LCP1 | DOCK2 |
| UNC93B1 | CCR1 |
| TLR2 | RNF19B |
| APOBEC3G | IFI30 |
| CD4 | SPI1 |
| C3 | SRGN |
| CD14 | LY96 |
| C2 | TLR2 |
| SLC11A1 | GPR65 |
| RNASE6 | PLEK |

HMOX1
NCF2
RAB32
HCLS1
FPR1
CD86
LY96
CASP4
CD300C
CMTM7
CYBA
LYN
GPSM3
C1QA
FCGR3A
OSCAR
HAVCR2
SPI1
HLA−DMA
LTBR
CD74
HLA−DRA
IFI30
NCF1
SLC16A3
GMFG
ALOX5
GPR65
RBM47
FTL
C1QC
CTSS
C1QB
SASH3
NPC2
CD300A
RAC2
ITGB2
CD53
SERPINB1
CTSC
FUCA1
SIGLEC9
ARPC1B
HLA−DMB
TYROBP
LAPTM5
VAMP8
SERPINA1
LAIR1
CD68
SLC7A7
S100A11

IQGAP1
GRN
FCER1G
HK3
CTSC
SLC11A1
MYO1G
A2M
C1R
SASH3
CTSZ
PTGER4
PDCD1LG2
SLC15A3
TNFRSF1B
SH2B3
MAN2B1
CSF2RB
THEMIS2
RAC2
B4GALT1
LYN
TMEM106A
DOK3
CD300C
APOBEC3C
CTSD
LCP2
LCP1
GNS
HEXB
CTSS
TCIRG1
SEC24D
NFAM1
ARPC1B
C1S
LAIR1
TLR1
CD300A
LTBR
ITGB2
LAPTM5
PYGL
CD4
CD68

FCGR2A
CTSZ
FCER1G
