# Supplementary material for: CTSB is a negative prognostic biomarker and therapeutic target associated with immune cells infiltration and immunosuppression in gliomas
Source: Sci Rep. 2022 Mar 11;12:4295. doi: 10.1038/s41598-022-08346-2 (PMC8917123; doi:10.1038/s41598-022-08346-2)
Supplement: Supplementary file 7 — Supplementary Information 7. [file 41598_2022_8346_MOESM7_ESM.pdf]

Supplementary Table S3. Inflammatory response-related genes and metagenes. There are 104 inflammatory genes which are divided into seven metagenes.

Genes	Metagenes
C1QB	HCK
C1QA	HCK
AIF1	HCK
LST1	HCK
DOCK2	HCK
LAPTM5	HCK
TYROBP	HCK
MS4A4A	HCK
MS4A6A	HCK
CD163	HCK
ITGB2	HCK
SLC7A7	HCK
LAIR1	HCK
HCK	HCK
TFEC	HCK
IFI30	HCK
MNDA	HCK
FCER1G	HCK
RNASE6	HCK
SLCO2B1	HCK
CCR1	HCK
IGSF8	IgG
ISLR2	IgG
IGSF21	IgG
IGSF1	IgG
IGSF22	IgG
IGDCC3	IgG
IGHD	IgG
IGSF11	IgG
IGSF5	IgG
IGSF6	IgG
IFIT1	Interferon
IFIT3	Interferon
IFI44L	Interferon
OAS3	Interferon
MX1	Interferon
RSAD2	Interferon
IFI44	Interferon
OAS2	Interferon
OAS1	Interferon
CD2	LCK
GZMK	LCK
GZMA	LCK
CD3D	LCK
CD53	LCK
LCK	LCK

ARHGAP15	LCK
CCL5	LCK
GMFG	LCK
SELL	LCK
STAT4	LCK
SAMSN1	LCK
RAC2	LCK
HCLS1	LCK
CCR7	LCK
PIK3CD	LCK
CORO1A	LCK
CD48	LCK
IL2RG	LCK
SH2D1A	LCK
SLAMF1	LCK
IL7R	LCK
INPP5D	LCK
KLRK1	LCK
FGL2	LCK
IRF8	LCK
SELPLG	LCK
IL10RA	LCK
SLA	LCK
CCR2	LCK
CSF2RB	LCK
HLA-E	MHC-I
HLA-H	MHC-I
HLA-B	MHC-I
HLA-J	MHC-I
HLA-F	MHC-I
HLA-G	MHC-I
HLA-A	MHC-I
HLA-C	MHC-I
HLA-L	MHC-I
HLA-DRB1	MHC-II
HLA-DRB5	MHC-II
HLA-DRB3	MHC-II
HLA-DPA1	MHC-II
HLA-DRA	MHC-II
HLA-DQA1	MHC-II
HLA-DQA2	MHC-II
HLA-DMA	MHC-II
HLA-DOA	MHC-II
HLA-DRB4	MHC-II
HLA-DMB	MHC-II
HLA-DQB1	MHC-II
HLA-DPB1	MHC-II
HLA-DQB2	MHC-II
CD74	MHC-II
PTPRC	MHC-II
HLA-DOB	MHC-II
HLA-DPB2	MHC-II
TAP1	STAT1

STAT1	STAT1
CXCL10	STAT1
CXCL11	STAT1
GBP1	STAT1
CXCL9	STAT1
