# Supplementary material for: CTSB is a negative prognostic biomarker and therapeutic target associated with immune cells infiltration and immunosuppression in gliomas
Source: Sci Rep. 2022 Mar 11;12:4295. doi: 10.1038/s41598-022-08346-2 (PMC8917123; doi:10.1038/s41598-022-08346-2)
Supplement: Supplementary file 8 — Supplementary Information 8. [file 41598_2022_8346_MOESM8_ESM.pdf]

Supplementary Table S4. Detailed information about the immune cell specific marker genes. There are some specific marker genes of CD4+ T cells, regulatory T cells (Tregs), CD8+ T cells, tumor-associated macrophages (TAMs), myeloid-derived suppressor cells (MDSCs) and neutrophils (NEUT).

Cells	Markers
Macrophages	CD14
Macrophages	HLADRA
Macrophages	CD312
Macrophages	CD115
Macrophages	CD163
Macrophages	CD204
Macrophages	CD301
Macrophages	CD206
Neutrophils	CD11b
Neutrophils	CD16
Neutrophils	CD66b
Neutrophils	ELANE
MDSCs	CD14
MDSCs	CD16
MDSCs	CD33
MDSCs	ARG1
CD8T	CD3E
CD8T	CD8A
Tregs	CD3E
Tregs	CD4
Tregs	CD25
Tregs	FOXP3
CD4T	CD3E
CD4T	CD4
